# Epidemiology of obesity and high blood pressure among school-age children from military families: the largest report from our region

**DOI:** 10.1186/s12887-023-03839-z

**Published:** 2023-01-23

**Authors:** Banafshe Dormanesh, Peyman Arasteh, Roya Daryanavard, Maryam Mardani, Meysam Ahmadi, Hamed Nikoupour

**Affiliations:** 1 Department of Pediatric, AJA University of medical sciences, Tehran, Iran; 2 AJA University of Medical Sciences, Tehran, Iran; 3 Shiraz University of Medical Sciences, Shiraz, Iran

**Keywords:** Obesity, High blood pressure, Military family, Children

## Abstract

**Background:**

For the first time, we aimed to determine the epidemiology and associated factors of obesity and hypertension among children of military families in our region.

**Methods:**

In this multi-centered study, children between the ages of 5 to 12 years old, entered the study. Data on baseline and clinical characteristics, history of disease and anthropometric measurements, were collected.

**Results:**

Among 504 children, 44.2% were males. Mean (SD) age of participants was 7.9 ± 1.9 years. Overall, 5% were obese and 9.9% were overweight. In total, 16.3% had elevated BP, 12.5% had stage one and 0.2% had stage two hypertension.

Age (beta = 0.306, OR = 1.35, 95% CI:1.14—1.61), obesity/overweight (OR = 5.58, 95% CI:2.59—12.0), history of hypertension in mother (OR = 43.24, 95% CI:5.99—312.11), low birth weight (OR = 7.96, 95% CI:2.59—12.0), physical activity (OR = 0.27, 95% CI:0.10—0.72), and consumption of fast food more than once a week (OR = 3.36, 95% CI:1.82—6.19), were associated with risk of hypertension.

Furthermore, age (beta = 0.346, OR = 1.41, 95% CI:1.21—1.64), history of childhood obesity in the father (OR = 3.78, 95% CI: 1.77—8.06) and mother (OR = 2.44, 95% CI:1.07—5.56), and physical activity (OR = 0.27, 95% CI:0.11—0.66), were associated with obesity.

**Conclusion:**

Age, obesity/overweight, history of hypertension in the mother, birth weight, physical activity, and consumption of fast food, were associated with risk of hypertension. Moreover, age, history of childhood obesity in parents, and physical activity, were associated with obesity.

Furthermore, we found that school-age children in military families have higher rates of hypertension and overweight compared to other reports from our region.

**Supplementary Information:**

The online version contains supplementary material available at 10.1186/s12887-023-03839-z.

## Introduction

Obesity is one of the most prevalent health issues that exists in the world today, the rate of which has had an increasing trend in many countries up to recent years [1]. This increasing trend is specially more pronounced within the pediatric population [2]. Obesity is associated with different diseases such as arthritis, cardiac problems, chronic pain, diabetes, and etc. [3]. It is associated with high blood pressure (BP), which itself is an important cause for cardiovascular disease, and cardiovascular diseases are the main causes of mortality worldwide [4, 5]. Childhood obesity is influenced by multiple factors, including genetic, behavioral and environmental determinants, among which behavioral factors play an important role on the development of obesity among pediatrics [1, 6].

Military families have specific and unique circumstances with regard to their health, considering different stressors which they are exposed to [7]. Due to the occupation of their parents, children in these families are faced with different conditions that may maliciously affect their mental health throughout their life-time compared to children in the general population. Accordingly, different studies have shown that suicidal ideation and stress is much more prevalent among military family members compared to their counterparts from a general population [8–10]. Moreover, diseases related to stress and depression, more specifically metabolic diseases, are more prevalent in this specific population. To this regard, obesity and high BP are directly linked to stress and depression [11].

On the other hand, data on the health conditions of military families are largely scarce and missing, especially from the Middle East.

Considering the specific characteristics of military families, in this study, we aimed to determine the epidemiology of obesity and hypertension among school-age children in military family. Furthermore, we determined the associated factors with obesity and high BP in this population.

## Patients and methods

### Study design

This is a multi-centered study conducted in three pediatric departments affiliated to AJA University of Medical Sciences, Tehran, Iran and Shiraz Artesh Hospital, Shiraz, Iran.

All patients between the ages of 5 to 12 years old who referred to our centers during a one-year period from March 21, 2021 to March 21, 2022 were included in this study. All children with chronic or systemic diseases (including musculoskeletal disorders, genetic diseases, neurologic, rheumatologic diseases, and etc), those with congenital diseases and those with malnutrition, were excluded from the study.

After referral of each patient to our centers, baseline information was obtained and each individual or their legal guardians were questioned regarding any diseases. After physical examination they were included in the study.

### Measurements

For measurement of BP in the pediatric population, a specific sphygmomanometer (Erka Perfect Aneroid, Germany) cuff was selected to be 0.45 to 0.55 of the child’s arm. The bladder of the BP cuff was also selected in a way to cover 80 to 100% of the circumference of the arm. Accordingly, for children who were overweight and obese, a larger BP device was selected [12–14].

Blood pressure was measured from the right arm and all participants were asked to rest for 5–10 minutes before any measurement was done. Three consecutive measurements were done for each individual. After a minimum of 5 minutes rest between each measurement, the mean measurement was considered that individuals’ BP. For the measurement, a stethoscope was placed on the brachial artery and the pressure of the sphygmomanometer was raised up to 20–30 mmHg more than the point where the radial pulse was undetectable [15].

Height was measured using a stadiometer (Seca 767, Japan) to the nearest 0.1 cm, furthermore weight was measured to the nearest 0.1 kg using a digital scale (Seca 767, Japan).

### Variables

blood pressure classification was done using the criteria defined by the American Academy of Pediatrics (AAP) for the screening and management of hypertension among children and adolescents [15]. Accordingly, among children between 5 and 13 years old, those with a BP less than the 90th percentile are considered normal; those with a BP of ≥90th and < 95th percentile or a BP ≥ 120 mmHg and < 130/80 mmHg are considered to have elevated BP. Those with a BP ≥ 95th percentile to BP < 95th percentile + 12 mmHg or a BP of 130/80 to 139/89, were considered as stage one hypertension, and those with a BP of ≥95th percentile + 12 mmHg or BP ≥140/90 mmHg were considered as stage two hypertension.

BMI was calculated by dividing weight (Kg) by the squared of height (m^2^). BMI classification was done using the CDC criteria. Accordingly, those with a BMI < 5th percentile are underweight, those with a BMI between the 5th and 85th percentile are normal, those between the 85th percentile and < 95th percentile are overweight, and those with a BMI ≥95th percentile are considered obese [16, 17].

Birth weight was categorized according to the WHO criteria. Low birth weight was defined as less than 2500 g. Normal birth weight was considered between 2500 and 4000 g and high birth weight was considered ≥4000 g [18].

Weekly fast-food intake and physical activity level were measured using a predesigned questionnaire and by direct questioning of both parents.

Weekly fast-food intake was categorized as: less than once a week (consuming less than one meal in a week), 1–3 times a week (consuming one to three meals of fast-food in any given week), more than 3 times a week (consuming more than three meals of fast-food in any given week) and never using fast-food.

Physical activity was assessed qualitatively using a self-report method. Categories were as followed: no activity (considered having no extra activity, other than normal daily activity), low level activity (having little extra activity), moderate activity (having extra activity similar to other children), high level of activity (perceived as having above average activity), and very high activity (such as those with ADHD).

### Statistical analysis

Data was analyzed using the Statistical Package for Social Sciences (SPSS Inc., Chicago, Ill., USA) software for windows, version 27. Data of quantitative variables with a normal distribution was compared between two groups using the independent T-Test. Data related to qualitative variables was compared between groups using the Chi-square test or the Fisher’s Exact test. Data is reported as means and standard deviations (SD) or median and interquartile range (IQR), where appropriate for quantitative variables, and as frequency and percentage for qualitative variables. A binary logistic regression model was used to estimate the associated factors with risk of hypertension and obesity in this population. A *p*-value of less than 0.05 was considered statistically significant.

## Results

During the study period, a total of 504 children entered the study. Among which, 44.2% (*n* = 223) were males. The mean (SD) age of participants was 7.9 ± 1.9 years. The mean BMI in the population was 17.0 ± 3.7 kg/m^2^.

Overall, 5% of the study population were obese and 9.9% were overweight. On the other hand, 5% were underweight.

Overall, 16.3% of the population had elevated BP, 12.5% had stage one hypertension and 0.2% had stage two hypertension. Only 0.8% of our study population had a positive history for high BP. Overall, 12.3 and 1.8% had a positive family history of hypertension among their fathers and mothers, respectively. None of the study participants had a positive history of hypertension among their siblings.

In our population, 20.8%, of the children had a premature birth.

Assessing the amount of weekly fast-food intake, showed that the majority of families consumed fast-food less than once a week (55.6%) and between 1 to 3 times (30.8%) a week.

A qualitative assessment of physical activity, showed that 22.5, 25.4, 36.4% had either no, low level or moderate level physical activity, respectively (Table 1).

**Table 1:** Comparison of baseline and clinical characteristics between males and females among school-age children from military families

Variables		Male	Female	***p***-value	Overall
Age - yrs	Mean (SD)	7.9 ± .2.0	7.9 ± 1.8	0.94	7.9 ± 1.9
BMI - kg/m2	Mean (SD)	16.9 ± 3.8	17.1 ± 3.6	0.62	17.0 ± 3.7
BMI classification - no.()	Underweight	10 (4.5)	15 (5.3)	0.92	25 (5)
	Normal weight	178 (44,1)	226 (55.9)		404 (80.2)
	Overweight	24 (10.8)	26 (9.3)		50 (9.9)
	Obese	11 (44)	14 (5)		25 (5)
Blood pressure status - no.(%)	Normal	153 (68.6)	205 (71)	0.55	358 (71)
	Elevated BP	40 (17.9)	42 (14.9)		82 (16.3)
	Stage 1	30 (13.5)	33 (11.7)		63 (12.5)
	Stage 2	0	1 (0.4)		1 (0.2)
History of high BP - no.(%)	Yes	2 (0.9)	2 (0.7)	0.81	4 (0.8)
	No	221 (99.1)	279 (99.3)		500 (99.2)
High BP in father - no.(%)	Yes	37 (16.6)	25 (8.9)	0.01	62 (12.3)
	No	186 (83.4)	256 (91.1)		442 (87.7)
High BP in mother - no.(%)	Yes	2 (0.9)	7 (2.5)	0.31	9 (1.8)
	No	221 (99.1)	274 (97.5)		495 (98.2)
High BP in siblings - no.(%)	Yes	0	0		0
	No	223 (44.2)	281 (55.8)	> 0.99	100
History of childhood obesity in father - no.(%)	Yes	29 (13)	42 (14.9)	0.60	71 (14.1)
	No	194 (87)	239 (85.1)		433 (85.9)
History of childhood obesity in mother - no.(%)	Yes	28 (12.6)	37 (13.2)	0.83	65 (12.9)
	No	195 (87.4)	244 (48.4)		439 (87.1)
Premature birth - no.(%)	Yes	45 (20.2)	60 (21.4)	0.74	105 (20.8)
	No	178 (79.8)	221 (78.6)		399 (79.2)
Weekly fast food use - no.(%)	Less than once	117 (52.5)	163 (58)	0.43	280 (55.6)
	1–3 times	76 (34.1)	79 (28.1)		155 (30.8)
	More than 3 times	5 (2.2)	4 (1.4)		9 (1.8)
	Never	25 (11.2)	35 (12.5)		60 (11.9)
Physical activity - no.(%)	No activity	13 (6)	97 (35.7)	< 0.001	110 (22.5)
	Low level	29 (13.4)	99 (36.4)		128 (25.4)
	Moderate	113 (52.3)	72 (26.5)		185 (36.7)
	High activity	59 (27.3)	4 (1.5)		63 (12.5)
	Very high activity	2 (0.4)	0		2 (0.4)

*BMI* body mass index, *BP* blood pressure, *SD* standard deviation, *IQR* interquartile range

Comparison between male and female children showed that, males had a higher rate of a positive history of hypertension among their fathers (16.6% vs. 8.9%; *p* = 0.01) and a higher rate of individuals with high and moderate level physical activity (27.3% vs. 1.5 and 52.3% vs. 26.5%, respectively; *p* < 0.001) (Table 1).

In a separate analysis, we found that 30% of overweight children had stage 1 hypertension and 40 and 4% of obese individuals had stage 1 and stage 2 hypertension, respectively.

In the multivariate analysis, age (beta = 0.306, OR = 1.35, 95% CI: 1.14—1.61), obesity/overweight (OR = 5.58, 95% CI: 2.59—12.0, compared to individuals with normal weight), positive history of hypertension in mother (OR = 36.99, 95% CI: 5.37—254.66), low birth weight (OR = 7.96, 95% CI: 2.59—12.0, compared to those with normal birth weight), moderate physical activity compared to low physical activity (OR = 0.27, 95% CI: 0.10—0.72), and consumption of fast food more than once a week compared to less than once a week (OR = 3.36, 95% CI: 1.82—6.19), were associated with hypertension (Table 2).

**Table 2:** Associated factors with hypertension among school-age children of military families

Variables		beta	Odd ratio	95% CI
Age		0.306	1.38	1.17—1.64
Sex	Male	–	Reference	
	Female	–	0.57	0.24—1.34
BMI	Normal	–	Reference	
	Obese or overweight	–	5.58	2.59—12.0
Positive history of High BP in father			2.39	0.96—5.94
Positive history of High BP in mother		–	36.99	5.37—254.66
Birth weight	Normal	–	Reference	
	Low birth weight	–	7.96	3.19—19.85
Physical activity	Low activity	–	Reference	
	Moderate activity	–	0.27	0.10—0.72
	High activity	–	0.71	0.25—1.97
Weekly fast food consumption	Less than once a week	–	Reference	
	More than once a week	–	3.36	1.82—6.19

*BMI* body mass index

Furthermore, age (beta = 0.346, OR = 1.41, 95% CI: 1.21—1.64), history of childhood obesity in the father (OR = 3.78, 95% CI: 1.77—8.06), history of childhood obesity in the mother (OR = 2.44, 95% CI: 1.07—5.56), and moderate physical activity compared to low physical activity (OR = 0.27, 95% CI: 0.11—0.66), were associated with obesity in our population (Table 3).

**Table 3:** Associated factors with obesity among school-age children of military families

Variables		beta	Odd ratio	95% CI
Age		0.346	1.41	1.21—1.64
Sex	Male	–	Reference	–
	Female	–	0.53	0.21—1.29
Positive history of High BP in father		–	1.76	0.78—3.95
Positive history of High BP in mother		–	0	0
History of childhood obesity in father		–	3.78	1.77—8.06
History of childhood obesity in mother		–	2.44	1.07—5.56
Birth weight	Normal	–	Reference	–
	Low birth weight	–	0	0
Physical activity	Low activity	–	Reference	–
	Moderate activity	–	0.27	0.11—0.66
	High activity	–	1.06	0.40—2.81
Weekly fast food consumption	Less than once a week	–	Reference	–
	More than once a week	–	1.12	0.61—2.06

*BMI* body mass index

Associated factors with obesity among male and female children from military families are shown in Table - S1 and Table - S2, respectively (Table-S1) (Table-S2).

Moreover, associated factors with risk of high blood pressure among male and female children from military families are shown in Table - S3 and Table - S4, respectively (Table-S3) (Table-S4).

## Discussion

In this study we aimed to evaluate the epidemiology of obesity and hypertension among a sample of children from military families. To the best of the authors’ knowledge, this is the largest report among school-age children of military families from the Middle East, which provides invaluable information.

Overall, 9.9 and 5% of the population were overweight and obese, respectively. This is comparable with a study by Mirmohammadi et al. [19] who reported on a large population of children between 7 and 18 years old. They found that 9.2 and 3.2% of individuals were overweight and obese, respectively. In a systematic review, Fakhri and colleagues [20], found that among a total of 93 studies, the rate of obesity and overweight in the pediatric population in Iran was 7 and 12%, respectively. In another systematic review by Kelishadi and colleagues [21], among a total of 27 studies from 2005 to 2010, authors reported the prevalence of obesity and overweight to be 7 and 11% among 7–11 year old children. Compared to the aforementioned reports from the normal population, school-age children in military families do not seem to have higher rates of obesity, although do have higher rates of overweight individuals. In another study from the US, among military families [22], authors evaluated a large number of children between 2 and 18 years old and compared them to their civilian counterparts. Accordingly, in their study the rate of overweight and obesity was 27 and 11.6%. They found that children from military families had lower rates of individuals who were overweight and obese compared to the normal population.

In a systematic review, Akbari et al. [23] evaluated hypertension among children between 3 and 18 years old. Among a total of seventeen relevant studies, they reported the prevalence of hypertension to be 8.9%. Compared to that of our population, we recorded higher rates of individuals with risk of hypertension (12.7% vs. 8.9%). In a similar study in the US [24], authors evaluated children between 4 to 17 years old from a large military academic center. Among a total of 3941 individuals, they found that 17% were obese. They also found 31% of obese children to have hypertension. To this regard, their results were similar to that ours. On the other hand, in the same study their obesity rates were higher compared to that of our population (5% vs. 17%).

A reason for the higher rates of individuals with risk of hypertension and individuals who were overweight in our population compared to other reports from the general population maybe attributed to a higher stress level among military families [9, 10], which has been linked to higher rates of metabolic disease [6, 11]. Although this would require validation by future studies. Furthermore, the stress and mental strain on military families maybe more pronounced in the Middle East region due to ongoing regional conflicts and war.

Another factor may be attributed to the high rate of obesity and overweight among adults in the military (parents of the children in military families). As one study [25] found a higher rate of obesity and overweight in military personnel compared to the general population in our region and this may be affecting children in these families, considering that studies have found that obesity in parents is related to obesity/overweight in children [26, 27].

Other factors which may have contributed to this, such as differences in dietary intake between children in military families and their counterparts from non-military families, require further studies in the future.

Interestingly in our study, out of the 12.7% that either had stage 1 or 2 hypertension, only 0.8% were previously diagnosed with high BP. This shows that only one in every fifteen individuals among school-age children in military families is aware of their existing hypertension. This calls for an urgent need to increase and expand screening programs in this specific population.

We found that age is associated with risk of hypertension, in other words older children had higher rates of hypertension. This could be attributed to the increase in obesity rates with increasing age. Obesity and physical activity were other factors associated with hypertension which are already known factors.

One interesting factor we found to be associated with risk of hypertension in our study, was a positive history of hypertension in the mother. This finding was similar to that reported in the study by Pang et al. [27], in which they studied 1288 school-aged children, and found that a positive family history of hypertension was significantly associated with hypertension among children (OR = 1.96, 95%: 1.16—3.32).

Low birth weight was also associated with risk of hypertension among school-age children. In one study, authors evaluated 45,319 children between the ages of 6 and 18 years old [28]. Similar to that of our study, authors found low birth weight to be significantly associated with hypertension (OR = 1.29, 95%: 1.02—1.64) among girls.

With regard to obesity, we found that with increasing age, rates of obesity also increase. This is compatible with the report by the CDC as they found an increasing trend in childhood obesity with age [29].

History of obesity in both the mother and father was also associated with obesity in the child. This finding was similar to that reported in the study by Bahreynian et al. [26], in which boys (OR = 2.79, 95% CI: 2.44–3.20) and girls (OR = 3.46, 95% CI: 3.03–3.94) with obese parents had higher chances of being obese, compared to those with normal parents. As expected, physical activity was protective against childhood obesity. Although we did not find a significant relationship between high intensity physical activity and obesity and only normal level physical activity was associated with obesity in our study. This may be due to multiple reasons, first is the qualitative measure of physical activity which may have had an observer bias as parents were answering the questions regarding their children’s level of physical activity. Second is that some of these children may have had diseases such as ADHD (which was not diagnosed as part of our study) and some studies have shown that ADHD may be associated with obesity and overweight [30, 31].

As a large number of those who join the military in the future are children of military families, a high rate of metabolic disease will eventually have a negative impact on the recruitment of these individuals, accordingly early detection and treatment is of vital importance in this specific population.

This study was not without limitations. First was the ongoing pandemic related to COVID-19 which made patient visitation difficult during the study period. The study was conducted among a military population, thus access to more specific data was limited due to the nature of the study. We could not assess military rank as a factor in our multivariate models, considering that some studies have shown that military rank may affect childhood obesity [32]. We assessed physical activity using a qualitative approach and by direct questioning of the parents which is not an accurate measure compared to a quantitative tool of assessment. Although for large scale epidemiological studies, a self-report tool is usually used for assessment of physical activity which may be associated with bias due to reasons such as the sporadic short burst nature of activity among children (which makes the assessment of physical activity more difficult among children) [33]. Blood pressure measurement was done using a sphygmomanometer and although this has long been considered the gold standard method of BP measurement for epidemiologic studies [34], more accurate methods such as the 24-hour ambulatory blood pressure monitoring (ABPM) do exist. From another point of view, using an ABPM for epidemiologic studies with large sample sizes is difficult, especially in countries with limited resources.

Moreover, some studies have shown that BP when using a sphygmomanometer is usually higher compared to a ABPM and this should be considered when interpreting our results [35].

From another aspect, this limitation would not have affected our comparison with reports within the region, as most of the studies in our region [23] have used a sphygmomanometer for the measurement of BP.

On the other hand, this is the largest report on the metabolic status of school-age children from military families from our region.

## Conclusion

For the first time we evaluated the condition of obesity and risk of high blood pressure among school-age children from military families in our region.

Age, obesity/overweight, history of hypertension in the mother, birth weight, physical activity, and consumption of fast food, were associated with risk of hypertension. Moreover, age, history of childhood obesity in parents, and physical activity, were associated with obesity in this specific population.

Furthermore, we found that school-age children in military families have higher rates of hypertension and overweight compared to other reports from our region.

## Supplementary Information

**Additional file 1: Table S1.** Associated factors with obesity among male school-age children of military families.

**Additional file 2: Table S2.** Associated factors with obesity among female school-age children of military families.

**Additional file 3: Table S3.** Associated factors with high blood pressure among male school-age children of military families.

**Additional file 4: Table S4.** Associated factors with obesity among female school-age children of military families.

## Data Availability

The data that support the findings of this study are available but restrictions apply to the availability of these data, which were used under license for the current study, and so are not publicly available. Data are however available from the authors upon reasonable request and with permission of the primary author (first author). Authors and institutions may request the raw data by directly contacting the primary author at dr.dormanesh@yahoo.com.
