# Supplementary material for: Epidemiology of obesity and high blood pressure among school-age children from military families: the largest report from our region
Source: BMC Pediatr. 2023 Jan 23;23:37. doi: 10.1186/s12887-023-03839-z (PMC9868491; doi:10.1186/s12887-023-03839-z)
Supplement: Supplementary file 1 — Additional file 1: Table S1. Associated factors with obesity among male school-age children of military families. [file 12887_2023_3839_MOESM1_ESM.docx]

| **Table-S1.** Associated factors with obesity among male school-age children of military families. | | | | |
| --- | --- | --- | --- | --- |
| **Variables** |  | **beta** | **Odd ratio** | **95% CI** |
| Age |  | 0.354 | 1.42 | 1.14—1.7 |
| Positive history of High BP in father |  | - | 4.72 | 1.62—13.70 |
| Positive history of High BP in mother |  | - | 0 | 0 |
| History of childhood obesity in father |  | - | 2.11 | 0.63—6.99 |
| History of childhood obesity in mother |  | - | 1.19 | 0.30—4.66 |
| Birth weight | Normal | - | Reference | - |
|  | Low birth weight | - | 0 | 0 |
| Physical activity | Low activity | - | Reference | - |
|  | Moderate activity | - | 0.28 | 0.08—0.97 |
|  | High activity | - | 1.32 | 0.42—4.10 |
| Weekly fast food consumption | Less than once a week | - | Reference | - |
|  | More than once a week | - | 0.43 | 0.16—1.14 |
