# Supplementary material for: Epidemiology of obesity and high blood pressure among school-age children from military families: the largest report from our region
Source: BMC Pediatr. 2023 Jan 23;23:37. doi: 10.1186/s12887-023-03839-z (PMC9868491; doi:10.1186/s12887-023-03839-z)
Supplement: Supplementary file 2 — Additional file 2: Table S2. Associated factors with obesity among female school-age children of military families. [file 12887_2023_3839_MOESM2_ESM.docx]

| **Table-S2.** Associated factors with obesity among female school-age children of military families. | | | | |
| --- | --- | --- | --- | --- |
| **Variables** |  | **beta** | **Odd ratio** | **95% CI** |
| Age |  | 0.298 | 1.34 | 1.04—1.73 |
| Positive history of High BP in father |  | - | 0.66 | 0.12—3.54 |
| Positive history of High BP in mother |  | - | 0 | 0 |
| History of childhood obesity in father |  | - | 6.26 | 1.99—19.66 |
| History of childhood obesity in mother |  | - | 3.52 | 1.05—11.75 |
| Birth weight | Normal | - | Reference | - |
|  | Low birth weight | - | 0 | 0 |
| Physical activity | Low activity | - | Reference | - |
|  | Moderate activity | - | 0.09 | 0.01—0.82 |
|  | High activity | - | 0 | 0 |
| Weekly fast food consumption | Less than once a week | - | Reference | - |
|  | More than once a week | - | 2.13 | 0.83—5.45 |
