# Supplementary material for: Epidemiology of obesity and high blood pressure among school-age children from military families: the largest report from our region
Source: BMC Pediatr. 2023 Jan 23;23:37. doi: 10.1186/s12887-023-03839-z (PMC9868491; doi:10.1186/s12887-023-03839-z)
Supplement: Supplementary file 3 — Additional file 3: Table S3. Associated factors with high blood pressure among male school-age children of military families. [file 12887_2023_3839_MOESM3_ESM.docx]

| **Table-S3.** Associated factors with high blood pressure among male school-age children of military families. | | | | |
| --- | --- | --- | --- | --- |
| **Variables** |  | **beta** | **Odd ratio** | **95% CI** |
| Age |  | 0.53 | 1.69 | 1.20—2.40 |
| BMI | Normal | - | Reference |  |
|  | Obese or overweight | - | 9.65 | 1.92—48.53 |
| Positive history of High BP in father |  | - | 0.77 | 0.04—12.41 |
| Positive history of High BP in mother |  | - | 0 | 0 |
| History of childhood obesity in father |  | - | 12.67 | 0.71—224.73 |
| History of childhood obesity in mother |  | - | 0 | 0 |
| Birth weight | Normal | - | Reference | - |
|  | Low birth weight | - | 71.70 | 9.13—562.97 |
| Physical activity | Low activity | - | Reference | - |
|  | Moderate activity | - | 0.01 | 0.001—0.10 |
|  | High activity | - | 0.16 | 0.02—1.05 |
| Weekly fast food consumption | Less than once a week | - | Reference | - |
|  | More than once a week | - | 23.95 | 4.90—116.99 |
