# Supplementary material for: Epidemiology of obesity and high blood pressure among school-age children from military families: the largest report from our region
Source: BMC Pediatr. 2023 Jan 23;23:37. doi: 10.1186/s12887-023-03839-z (PMC9868491; doi:10.1186/s12887-023-03839-z)
Supplement: Supplementary file 4 — Additional file 4: Table S4. Associated factors with obesity among female school-age children of military families. [file 12887_2023_3839_MOESM4_ESM.docx]

| **Table-S4.** Associated factors with obesity among female school-age children of military families. | | | | |
| --- | --- | --- | --- | --- |
| **Variables** |  | **beta** | **Odd ratio** | **95% CI** |
| Age |  | 0.28 | 1.33 | 1.04—1.71 |
| BMI | Normal | - | Reference |  |
|  | Obese or overweight | - | 5.90 | 2.02—17.23 |
| Positive history of High BP in father |  | - | 2.16 | 0.55—8.51 |
| Positive history of High BP in mother |  | - | 14.35 | 1.84—111.39 |
| History of childhood obesity in father |  | - | 0.72 | 0.18—2.91 |
| History of childhood obesity in mother |  | - | 0.56 | 0.12—2.53 |
| Birth weight | Normal | - | Reference | - |
|  | Low birth weight | - | 4.73 | 1.26—17.75 |
| Physical activity | Low activity | - | Reference | - |
|  | Moderate activity | - | 0.69 | 0.21—2.27 |
|  | High activity | - | 0 | 0 |
| Weekly fast food consumption | Less than once a week | - | Reference | - |
|  | More than once a week | - | 1.81 | 0.76—4.31 |
| BMI: body mass index | | | | |
